# High Intensity High Volume Interval Training Improves Endurance Performance and Induces a Nearly Complete Slow-to-Fast Fiber Transformation on the mRNA Level

**DOI:** 10.3389/fphys.2018.00601

**Published:** 2018-05-29

**Authors:** Julian Eigendorf, Marcus May, Jan Friedrich, Stefan Engeli, Norbert Maassen, Gerolf Gros, Joachim D. Meissner

**Affiliations:** ^1^Institute of Sports Medicine, Hannover Medical School, Hannover, Germany; ^2^Clinical Research Center Hannover, Hannover Medical School, Hannover, Germany; ^3^Institute of Clinical Pharmacology, Hannover Medical School, Hannover, Germany; ^4^Institute of Sports Science, Leibniz University Hannover, Hannover, Germany; ^5^Molecular and Cell Physiology, AG Vegetative Physiology, Hannover Medical School, Hannover, Germany

**Keywords:** energy metabolism, interval training, myosin heavy chain, performance parameter, muscular aerobic capacity, systemic aerobic capacity

## Abstract

We present here a longitudinal study determining the effects of two 3 week-periods of high intensity high volume interval training (HIHVT) (90 intervals of 6 s cycling at 250% maximum power, P_max_/24 s) on a cycle ergometer. HIHVT was evaluated by comparing performance tests before and after the entire training (baseline, BSL, and endpoint, END) and between the two training sets (intermediate, INT). The mRNA expression levels of myosin heavy chain (MHC) isoforms and markers of energy metabolism were analyzed in M. vastus lateralis biopsies by quantitative real-time PCR. In incremental tests peak power (P_peak_) was increased, whereas $\dot{V}$O_2peak_ was unaltered. Prolonged time-to-exhaustion was found in endurance tests with 65 and 80% P_max_ at INT and END. No changes in blood levels of lipid metabolites were detected. Training-induced decreases of hematocrit indicate hypervolemia. A shift from slow MHCI/β to fast MHCIIa mRNA expression occurred after the first and second training set. The mRNA expression of peroxisome proliferator-activated receptor gamma coactivator 1α (PGC-1α), a master regulator of oxidative energy metabolism, decreased after the second training set. In agreement, a significant decrease was also found for citrate synthase mRNA after the second training set, indicating reduced oxidative capacity. However, mRNA expression levels of glycolytic marker enzyme glyceraldehyde-3-phosphate dehydrogenase did not change after the first and second training set. HIHVT induced a nearly complete slow-to-fast fiber type transformation on the mRNA level, which, however, cannot account for the improvements of performance parameters. The latter might be explained by the well-known effects of hypervolemia on exercise performance.

## Introduction

Sprint interval training has been predicted to be a potent and time-efficient means of improving endurance exercise performance (Coyle, 2005). This was based on a study demonstrating that sprint interval training can improve cycle endurance time significantly (Burgomaster et al., 2005). Extending this study, Gibala et al. (2006) could demonstrate that a low volume sprint interval training can elicit increases in exercise capacity, markers of oxidative capacity, resting muscle glycogen content, and buffering capacity, all of these changes being comparable to those induced by a high volume endurance training. Furthermore, in a subsequent study using a similar low volume high intensity interval sprint training, exercise capacity (maximal oxygen consumption, $\dot{V}$O_2peak_), and markers of oxidative capacity have been shown to increase along with other metabolic adaptations as seen after endurance training (Burgomaster et al., 2008). Notably, in the latter two studies the similar effects of sprint interval and endurance training on endurance performance were achieved despite large differences in training volume and time commitment. However, data about fiber type proportions or myosin heavy chain (MHC) isoform expression have not been presented in these studies.

In contrast, the effect of endurance training on muscle fiber type and MHC expression has been investigated in numerous studies. In a large number of studies, MHCIId/x has been shown to be down-regulated following increased physical activity, with MHCIIa being upregulated. The majority of experiments failed to demonstrate the full MHC shift of a fast-to-slow transformation (Mercier et al., 1999; Harridge, 2007). These findings led to the hypothesis that, at least in humans, fast-to-slow transformations require a significant challenge to the muscle (Harridge, 2007). In contrast, in animal models fast-to-slow transformations can be easily achieved, for example by low frequency electrostimulation (Pette and Vrbova, 1992). However, some examples of exercise-induced fast-to-slow transformations in humans do exist, and, interestingly, training regimen using endurance and high intensity interval training (HIT) were among them (Howald et al., 1985; Simoneau et al., 1985; Linossier et al., 1993; Russell et al., 2003a,b). Sprint training in general is associated with a decreased proportion of slow and an increased proportion of fast fibers (Abernethy et al., 1990). For example, a running sprint HIT led to a decrease in slow type I and an increase in fast type II fiber area (Dawson et al., 1998). In the wake of a high intensity cycle sprint training (Jansson et al., 1990), the proportion of type I fibers decreased while the proportion of type IIA increased and that of type IID remained unchanged, demonstrating again that high intensity sprint training can lead to a slow-to-fast transformation. In contrast, a 5-s sprint cycle training as presented by Linossier et al. (1993) induced a fast-to-slow fiber type shift with an increase in the number of type I and a decrease in type IID fibers, while the number of type IIA fibers remained unaltered. Strikingly, activities of two marker enzymes of oxidative energy metabolism did not change in this 5-s sprint training, while the activities of two glycolytic marker enzymes showed an increase. The metabolic changes seen in this study do not appear to be consistent with the effects on MHC isoform expression, and therefore all changes together did not constitute a classical fast-to-slow transformation. Nevertheless, the study provides an interesting training design that might be used to induce increases in MHCI/β gene expression. The study also seems to indicate that the elements of exercise-induced fiber transformations are not inevitably all consistent with either “slow-to-fast” or “fast-to-slow,” nor must a transformation be complete. Indeed, a metabolic transformation is often achieved more easily and can occur without changes in MHC expression. This is compatible with the finding that fast fibers show a high degree of adaptability of their oxidative capacity in response to endurance training (Abernethy et al., 1990).

The effects of endurance exercise on oxidative capacity are thought to be mediated by the transcriptional coactivator peroxisome proliferator-activated receptor gamma (PPARγ) coactivator 1α (PGC-1α) (Qaisar et al., 2016). PGC-1α is crucial regulator of mitochondrial biogenesis and oxidative energy metabolism (Puigserver and Spiegelman, 2003; Arany, 2008) and has been reported to be activated in response to endurance exercise (Qaisar et al., 2016). Furthermore, several studies have demonstrated that HIT also induces an up-regulation of PGC-1α (Psilander et al., 2010; Little et al., 2011; Cochran et al., 2014). Nevertheless, based on studies in PGC-1α knockout mice, it has been questioned whether PGC-1α is necessary for the majority of adaptive responses to exercise (Egan and Zierath, 2013).

Motivated by the finding of a HIT-induced shift from fast to slow fibers by Linossier et al. (1993), we developed for this study a 6-s sprint high intensity high volume interval training study (HIHVT; modification of the protocol of Schrader et al., 2016) designed to answer the following questions. First: Will the 6-s sprint HIHVT induce an increase in the expression of slow MHCI/β associated with decreased expression of fast MHC isoforms? Second: Is the possible shift toward MHCI/β expression accompanied by increases in the expression of markers of oxidative energy metabolism? Third: Do muscular adaptations encompass decreases in glycolytic marker expression, thus constituting a complete HIHVT-induced fast-to-slow fiber type transformation?

Contrary to the results of Linossier et al. (1993), the present 6-s sprint HIHVT led to a slow-to-fast shift in MHC expression that was accompanied by a decrease in PGC-1α and citrate synthase (CS) mRNA and a trend toward a decreased mRNA expression of another marker of oxidative energy metabolism, hydroxyacyl-CoA-dehydrogenase (HADH), but no change in glycolytic marker glyceraldehyde-3-phosphate dehydrogenase GAPDH). The data therefore indicate a nearly complete HIHVT-induced slow-to-fast fiber type transformation on the mRNA level, with the exception of the marker of glycolytic energy metabolism. The observed changes in fiber type characteristics cannot explain the observed improvements in endurance performance. Thus, other factors seem to be responsible for these effects of HIHVT.

## Methods

### Subjects

Eight healthy, male subjects (Table 1) were recruited for the study after their written informed consent had been given and their coagulation status had been tested. Normal coagulation status was an inclusion criterion. One subject could not complete the second training session, leaving *n* = 7 for the last investigated time point of the session. The training status of the subjects was heterogeneous, one was untrained and several others were recreational athletes. All of them had bicycling experience, three subjects were extensive cyclists. Subjects were asked to refrain from any specific diet during the study and keep their standard nutritional habits, with the exception that all of them were requested to observe a carbohydrate-rich diet on the 2 days before a test. Also, they were asked to refrain from sporting activities for 48 h before each test. All tests were performed on the same time of the day. The study procedures recorded in the study protocol were approved by the local ethics committee of Hannover Medical School.

**Table 1:** Subject characteristics.

**Height (cm)**	**Weight (kg)**	**Age (yr)**	**P_peak_ (W)**	**P_peakrel_ (W/kg)**
183 ± 3	77.7 ± 1.7	26.8 ± 2.1	308 ± 24	4.0 ± 0.3

**Figure 1:**
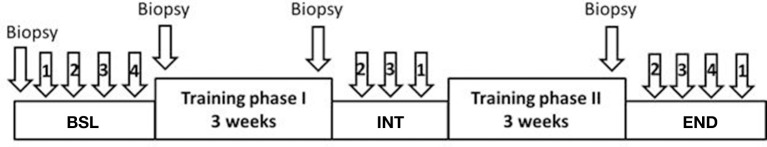
Schematic representation of training and testing sessions. Training phase I: first set of 9 training sessions in 3 weeks; training phase II: second set of 9 training sessions in 3 weeks. Testing sessions: baseline, BSL, before begin of training; intermediate, INT, week between the two training phases; endpoint, END, after end of training. 1: Incremental test (IT); 2: Wingate test (WT); 3: Endurance test 80% (ET80); 4: Endurance test 65% (ET65).

*Number of subjects: 8; P_peak_, peak power from the incremental test of the first testing session (see Figure 1); P_peakrel_, peak power/body weight*.

### Training and testing sessions

All training sessions started with 5 min at rest on the cycle ergometer, followed by a running-in for 2 min at 10 Watt (W), a 10 min warm-up at 50% maximum power (P_max_), a 45 min interval phase with 90 intervals of 6 s at 250% P_max_, each followed by a 24 s pause at 10 W, and a cool-down of 5 min at 50% P_max_. Subjects were asked to keep cadence at 70-90 min^−1^. Subjects performed three training sessions per week, resulting in 18 training sessions in 6 weeks. The training was interrupted after 3 weeks (first phase with a set of 9 training sessions) for 1 week for the intermediate (INT) testing procedures (Figure 1). Afterwards, training was resumed for another 3 weeks (second phase with a set of 9 training sessions). Additional testing sessions were scheduled before the first training session (baseline, BSL) and after the last (eighteenth) training session (endpoint, END) (see Figure 1 for set-up of testing sessions). In addition, during the second, eighth, and seventeenth training session several parameters were monitored (see below).

### Exercise performance tests

**Figure 2:**
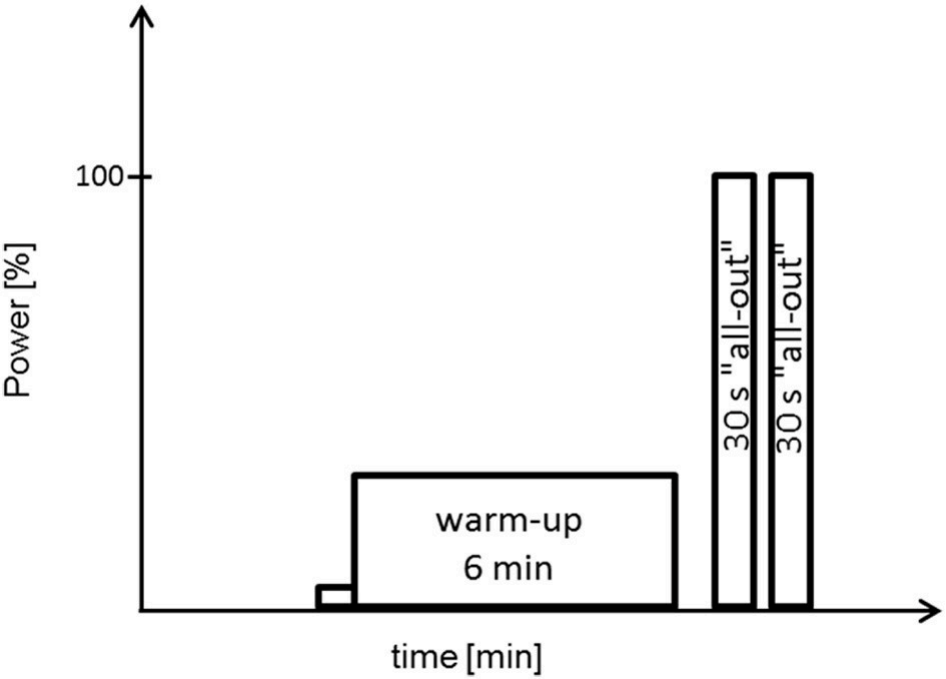
Schematic representation of a doubled Wingate-Test. Power output during the two Wingate-tests is set to 100%. One minute pause between warm-up and the two 30 s all-out sprints.

All tests and training sessions were performed on a Lode Excalibur cycle ergometer (Groningen, The Netherlands). Testing procedures included an incremental test (IT), two endurance tests (ET) and a doubled Wingate-Test (WT). The IT was used for assessing $\dot{V}$O_2peak_ and peak power (P_peak_). Subjects completed two ETs to exhaustion, one with 65% P_max_ (ET65) and another with 80% P_max_ (ET80). To test for sprint and regeneration capability subjects performed a doubled WT, i.e., two 30 s all-out sprints with 1 min pause in between (Figure 2).

Ventilation ($\dot{V}$E), oxygen consumption ($\dot{V}$O_2_), carbon dioxide release ($\dot{V}$CO_2_) and the respiratory exchange ratio (RER) were determined by spirometry using a Metalyzer 3b (Cortex, Leipzig, Germany).

### Blood parameters and metabolites

During WT, ET65, ET80, and selected (second, eighth, seventeenth) training sessions venous blood samples from an antecubital vein were drawn. EDTA or sodium-heparin was used for anticoagulation. Blood samples were immediately centrifuged at 4°C, plasma was aliquoted and deep frozen at −80°C for later analysis.

Glycerol (Gly) concentration [Gly] was determined using a UV-method (R-Biopharm AG, Darmstadt, Germany). Triglyceride concentration [Tri] was calculated from the difference of the concentrations of free Gly [fGly] and total Gly [tGly]. For determination of [tGly], probes underwent alkaline hydrolysis to release esterified Gly.

FFA were analyzed using the NEFA-HR(2) enzymatic color Test and standards (Wako Chemicals GmbH, Neuss).

Venous blood samples for hematocrit measurements were drawn into heparinized micro hematocrit tubes (Brand GmbH & Co KG, Wertheim, Germany), immediately centrifuged and evaluated.

Capillary blood samples were drawn from an earlobe during all testing sessions. Earlobes were hyperaemised at least for 3 min before the first sample was drawn. Capillary blood drawn into 20 μl end-to-end capillaries was used to analyze glucose and lactate concentrations within the next 2 h. Analyses were performed with the Biosen S line analyzer (EKF Diagnostics, Barleben, Germany) including the glucose/lactate test set-up with hemolyzing, Glucose/Lactate system, multi standard and ReadyCon test solutions (EKF Diagnostics).

### Muscle biopsies

All muscle biopsies were taken from the left vastus lateralis muscle. After skin disinfection using iodine-containing solutions, the lower third of the muscle was covered with a sterile surgical sheet. Skin and muscle fascia were anesthetized with 2% lidocaine plus epinephrine to reduce bleeding (Xylocitin®, 2 ml containing 32.46 mg lidocaine and 0.02 mg epinephrine). A spring biopsy needle (Biopsy Handy 14G, Somatex, Teltow, Germany) was inserted through a small skin incision and moved forward through the fascia into the muscle to obtain muscle tissue pieces of 5–8 mm in length (~10 mg wet weight). The typical depth of sampling was 1.5–2 cm below the muscle fascia with little variation because the fascia serves as an important landmark during the procedure and the needle construction allows a standardized and controlled insertion including marks on the outside. The sampling was performed through the same fascia puncture in the lower third of the M. vastus lateralis, and the orientation of the needle was strictly pointed to the knee. Thus, repeated tissue samples were taken within close proximity. Each biopsied tissue piece was immediately snap frozen in liquid nitrogen and kept at −80°C until analysis. The wound was afterwards covered with adhesive bandage and a pressure bandage was applied for 60 min. The first biopsy was taken under resting condition in supine position at least 2–3 days before the first pretest. The other three biopsies were taken in sitting position (on the ergometer) immediately after the last training bout of the first, ninth, and eighteenth training session (the latter at the end of the first and of the second set of training sessions, respectively) (see Figure 1). For this purpose, all steps described above to prepare the subjects for biopsy taking were performed within the breaks during the last 15–20 cycles so that the biopsy needle could be inserted into the muscle immediately after the last sprint. Except for mild to moderate muscle pain on the following 2–3 days after the biopsy, volunteers experienced no adverse effects.

### Quantitative real-time PCR (qPCR)

Total RNA was isolated from muscle biopsies using the RNeasy Fibrous Tissue Mini Kit (Qiagen, Hilden, Germany; including Dnase I treatment) and subsequently the RNeasy MinElute Cleanup Kit (Qiagen) according to the manufacturer's instructions. A Micro-Dismembrator (B. Braun Melsungen AG, Melsungen, Germany) was used for tissue disruption and homogenization. A cDNA synthesis with integrated removal of genomic DNA contamination was performed with the QuantiTect Reverse Transcription Kit (Qiagen).

Analysis by qPCR was performed on a Rotor-Gene 2000 real-time PCR thermocycler (Qiagen, Hilden, Germany; program: 40 cycles of 95°C for 10 s followed by 60°C for 45 s) using Power SYBR Green Supermix (Applied Biosystems, Darmstadt, Germany) according to the manufacturer's instructions. Data were quantified using Rotor-Gene Q Series Software 1.7 (Qiagen). Efficiencies were calculated from the slope of template dilution curves with primers for genes of interest (MHC isoforms or genes of energy metabolism) and the reference gene (BSM or RPS12, respectively), and used for quantification of changes of transcript levels by the ΔΔCt-method (Livak and Schmittgen, 2001). The efficiency of all primer sets was between 95 and 105%.

### Primers

Human citrate synthase (CS; GenBank accession no. NM_004077.2)

forward primer: 5′-GCA GAA GGA AGT TGG CAA AG-3′
reverse primer: 5′-CGC GGA TCA GTC TTC CTT AG-3′

Human glyceraldehyde-3-phosphate dehydrogenase (GAPDH; GenBank accession no. NM_002046.5)

forward primer: 5′-AGA ACG GGA AGC TTG TCA TC-3′
reverse primer: 5′-GAC TCC ACG ACG TAC TCA GC-3′

Human hydroxyacyl-CoA-dehydrogenase (HADH; GenBank; accession no. NM_005327.4)

forward primer: 5′-TCC TGG CAA AAT CCA AAA AG-3′
reverse primer: 5′-AGT CTG TGC TGT GGA CAA CG-3′

Human β2-Mikroglobulin (B2M; GenBank accession no. NM_004048.2)

forward primer: 5′-TTC TGG CCT GGA GGC TAT C-3′
reverse primer: 5′-TCA GGA AAT TTG ACT TTC CAT TC-3′

Human myosin heavy chain I/β (MHCI/β; GenBank accession no. NM_000257.2)

forward primer: 5′-GGG CTT GAA TGA GGA GTA GC-3′
reverse primer: 5′-CCC AAG GAG CTG TTA CAC AG-3′

Human myosin heavy chain IIa (MHCIIa; GenBank accession no. NM_017534.5)

forward primer: 5′-ATG CCA TGG AAT GAC TGA AG-3′
reverse primer: 5′-TGC AAC AGG GTA GAA TAC AC-3′

Human myosin heavy chain IId/x (MHCIId/x; GenBank accession no. NM_005963.3)

forward primer: 5′-TCT AAC TGC TGA AAG GTG AC-3′
reverse primer: 5′-TCT CCA AAA GTC ATA AGT AC-3′

Human peroxisome proliferator-activated receptor gamma (PPARγ) coactivator 1α (PGC-1α; GenBank accession no. NM_013261.3)

forward primer: 5′-CAC TTG AGT CCA CCC AGA AA-3′
reverse primer: 5′-GAC ATC GAG TGT GCT GCT CT-3′

Human ribosomal protein S12 (RPS12; GenBank accession no. NM_001016.3)

forward primer: 5′-AAG GCA TAG CTG CTG GAG GTG
TAA-3′
reverse primer: 5′-AGT TGG ATG CGA GCA CAC ACA
GAT-3′

Human superoxide dismutase 2 (SOD2; GenBank accession no. NM_000636.2)

forward primer: 5′-AGG CAA ACT TCA ACA GCA AA-3′
reverse primer: 5′-GGA GAT TGG GTC TCA AGC AT-3′.

### Statistics

Statistical analyses were performed using SigmaPlot 11.0 (Systat Software Inc.) and GraphPad Prism 6 (GraphPad Software, Inc.). Figures and tables show mean values (MV) with standard error (SE). Statistical significance was set to *p* < 0.05. For statistical analyses of data obtained during the time course of BSL, INT and END testing, respectively, two way repeated measurement ANOVA was used. To correct for multiple comparisons Holm–Sidak *post-hoc* tests were applied after all ANOVA tests when significant interactions of two factors were detected. Additionally, one-way repeated measures parametric or non-parametric ANOVA was used. Homogeneous distribution of variances was tested for by the Levene Median test.

## Results

### Training

**Figure 3:**
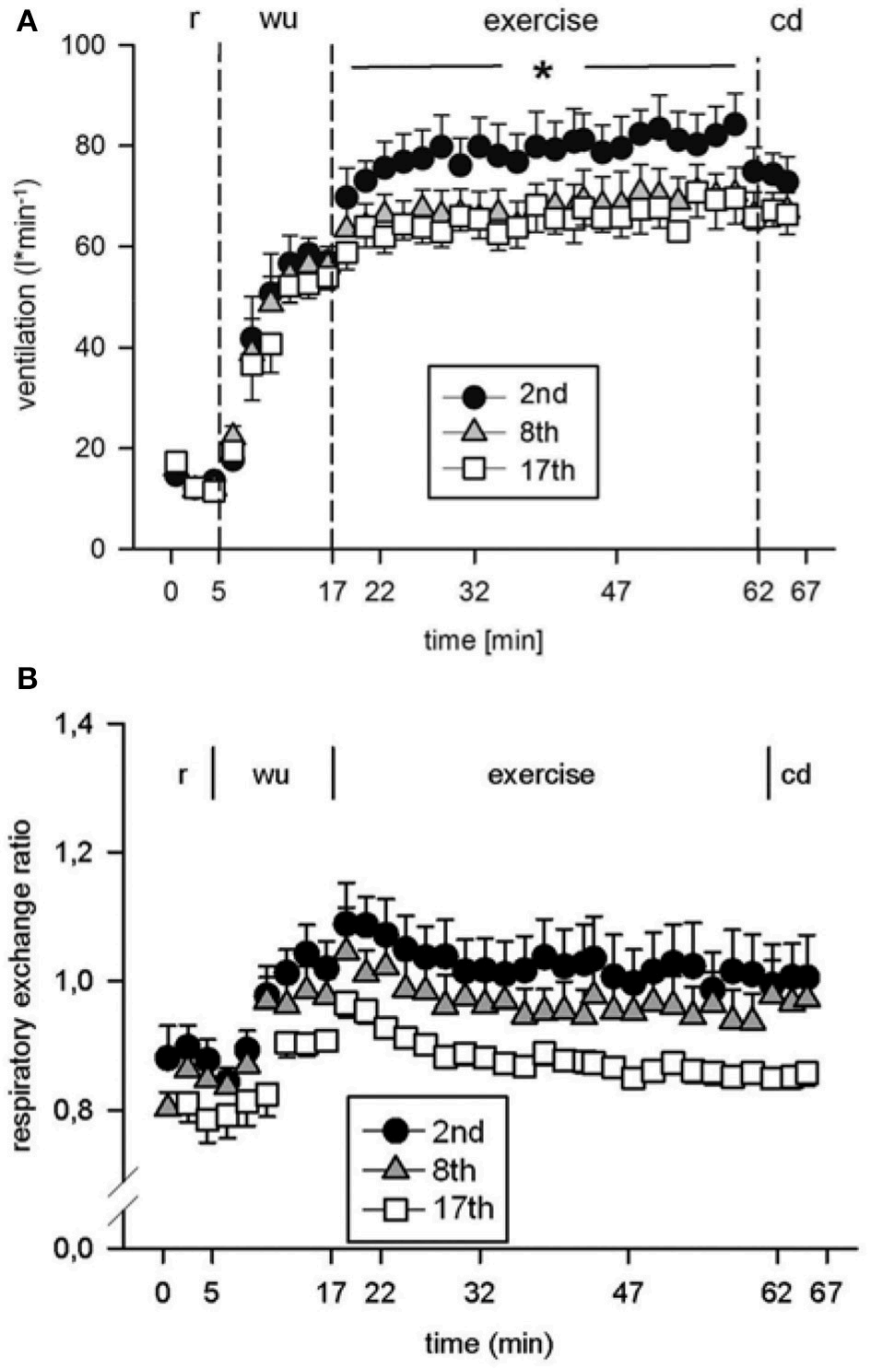
Analysis of **(A)** ventilation and **(B)** respiratory exchange ratio during the second (2nd), eighth (8th), and seventeenth (17th) training session of the high intensity high volume interval training (HIHVT). Data are presented as means ± SE. Minutes 0–5: rest (r) on the cycle ergometer; min 6–17: 2 min running-in at 10 Watt (W) followed by 10 min warm-up (wu) at 50% maximum power (P_max_); min 18–62: training session with 90 intervals of 6 s at 250% P_max_, each followed by a 24 s-pause at 10 W (exercise); min 63–67: cool-down (cd) at 50% P_max_. ^*^Significantly different (8th and 17th training session, respectively) from the 2nd training session, *p* < 0.05, at indicated period of time (min 18–62).

Eight subjects (Table 1) performed a high intensity high volume interval training (HIHVT) consisting of two sets of 9 training sessions (90 intervals of 6 s cycling at 250% maximum power, P_max_/24 s, per session). Each set was completed within 3 weeks, and 1 week was interposed between the two sessions. HIHVT was performed on a cycle ergometer (Figure 1). In the course of the HIT sessions, several parameters as described below were closely monitored. During the second, eighth, and seventeenth training session, ventilation ($\dot{V}$E) was significantly increased compared to rest (min 18–62 and 0–5, respectively, Figure 3A). During the eighth and seventeenth compared to the second training session, $\dot{V}$E was significantly lower at indicated time points (min 18–62). Respiratory parameters mean carbon dioxide release ($\dot{V}$CO_2mean_) and mean oxygen consumption ($\dot{V}$O_2mean_) did not show significant differences between the second, eighth, and seventeenth training session (Table 2), the latter indicating no changes in systemic aerobic capacity. Similarly, the mean respiratory exchange ratio (RER) was not significantly different between the second, eighth, and seventeenth training session (Table 2, Figure 3B).

**Table 2:** Performance, respiratory, and metabolic parameters during or after HIHVT training sessions.

	**P_mean_ (W)**	VO_2 mean_ (l^*^min^−1^)	VCO_2 mean_ (l^*^min^−1^)	**RER_mean_**
2nd	730 ± 60	2.33 ± 0.02	2.37 ± 0.02	1.03 ± 0.01
8th	730 ± 60	2.27 ± 0.02	2.18 ± 0.02	0.97 ± 0.01
17th	730 ± 60	2.39 ± 0.03	2.11 ± 0.02	0.88 ± 0.01
	Glucose_rest_ (mmol^*^l^−1^)	Glucose_end_ (mmol^*^l^−1^)	Lactate_rest_ (mmol^*^l^−1^)	Lactate_end_ (mmol^*^l^−1^)
2nd	5.58 ± 0.20	4.77 ± 0.34	1.04 ± 0.11	4.72 ± 0.70^*^
8th	5.09 ± 0.22	4.85 ± 0.32	0.93 ± 0.09	4.08 ± 0.51^*^
17th	5.18 ± 0.23	5.00 ± 0.30	0.98 ± 0.15	2.89 ± 0.25^*^
	Tri_rest_ (mmol^*^l^−1^)	Tri_end_ (mmol^*^l^−1^)		
2nd	1.12 ± 0.21	0.82 ± 0.13		
8th	1.46 ± 0.32	1.37 ± 0.43		
17th	1.60 ± 0.29	1.18 ± 0.20		

Performance and respiratory parameters during the second (2nd), eighth (8th), and seventeenth (17th) training session of HIHVT. Metabolic parameters at rest (rest) and at the end (end) of the second, eighth, and seventeenth training session of HIHVT. Data are presentedas means ± SE. P_mean_, mean power during 6 s training intervals ofthe indicated training session; RER_mean_, mean respiratory exchange ratio during 6s training intervals; Tri, triglycerides.

* Significantly different from Lactate_rest_, p < 0.01.

**Figure 4:**
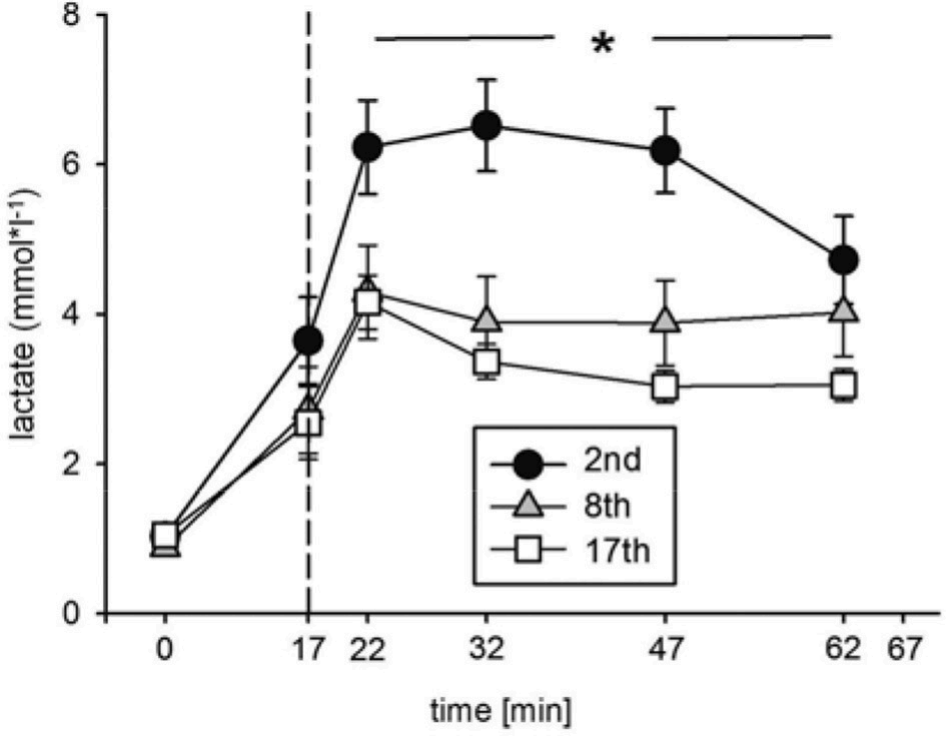
Analysis of blood lactate concentrations during the second (2nd), eighth (8th), and seventeenth (17th) training session of the HIHVT. Data are presented as means ± SE. Minutes 0–5: rest on the cycle ergometer; min 6–17: 2 min running-in at 10 Watt (W) followed by 10 min warm-up at 50% maximum power (P_max_); min 18–62: training session with 90 intervals of 6 s at 250% P_max_, each followed by a 24 sec-pause at 10 W; min 63–67: cool-down at 50% P_max_. ^*^Significantly different (8th and 17th training session, respectively) from the second training session, *p* < 0.01, at indicated points in time (min 18–62).

Blood lactate concentration ([Lac]) was significantly decreased at the indicated time points during the eighth and seventeenth compared to the second training session (Figure 4, Table 2). The highest [Lac], 6.5 mmol^*^l^−1^, was measured during the second training session. These data indicate improvements in energy metabolism elicited during the HIHVT exercise tests.

We next analyzed the effects of the HIHVT on substrate utilization. Significant differences in blood glucose concentrations [Glu] were neither found at the end of the second, eighth, and seventeenth interval training session in comparison to [Glu] at rest, nor between the second, eighth, and seventeenth interval training sessions (Table 2), indicating no HIHVT-induced changes in glucose levels.

**Figure 5:**
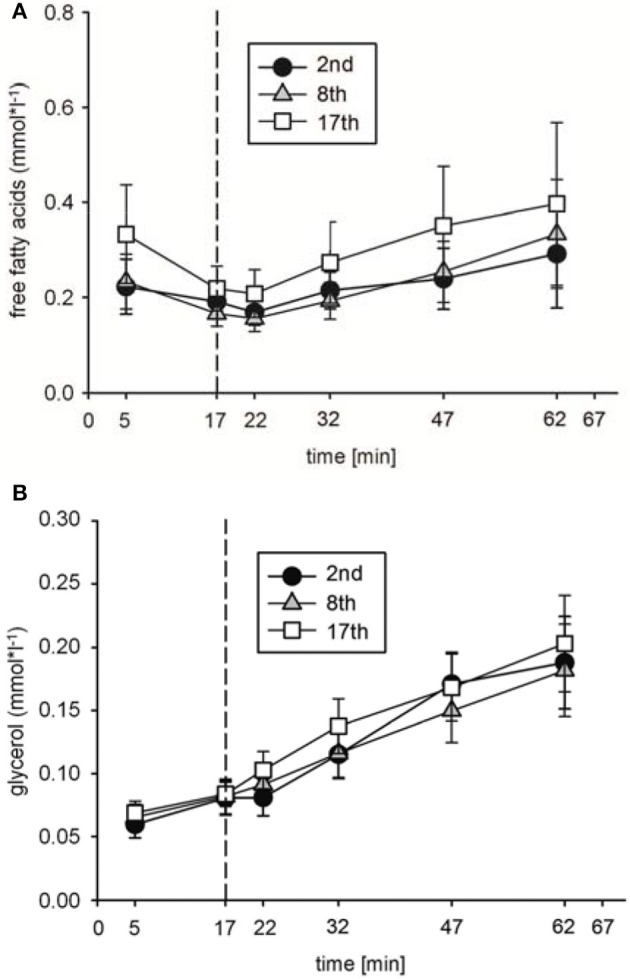
Analysis of **(A)** free fatty acid and **(B)** glycerol concentrations during the second (2nd), eighth (8th), and seventeenth (17th) training session of the HIHVT. Data are presented as means ± SE. Minutes 0–5: rest on the cycle ergometer; min 6–17: 2 min running-in at 10 Watt (W) followed by 10 min warm-up at 50% maximum power (P_max_); min 18–62: training session with 90 intervals of 6 s at 250% P_max_, each followed by a 24 s-pause at 10 W; min 63–67: cool-down at 50% P_max_.

To analyze possible effects of HIHVT on lipid metabolism, concentrations of plasma free fatty acids [FFA], glycerol [Gly], and triglyceride [Tri] as parameters of lipid metabolism were determined. [FFA] (Figure 5A) and [Gly] (Figure 5B) did neither change significantly between rest and indicated time points of the second, eighth, and seventeenth interval training session nor between the second, eighth, and seventeenth interval training sessions. Moreover, no significant changes in [Tri] were found at the end of the second, eighth, and seventeenth interval training session in comparison to the respective [Tri] at rest, nor between the sessions at the end of the training (Table 2). The data indicate no changes in lipid metabolism during the HIHVT.

### Testing sessions

#### Exercise testing

**Figure 6:**
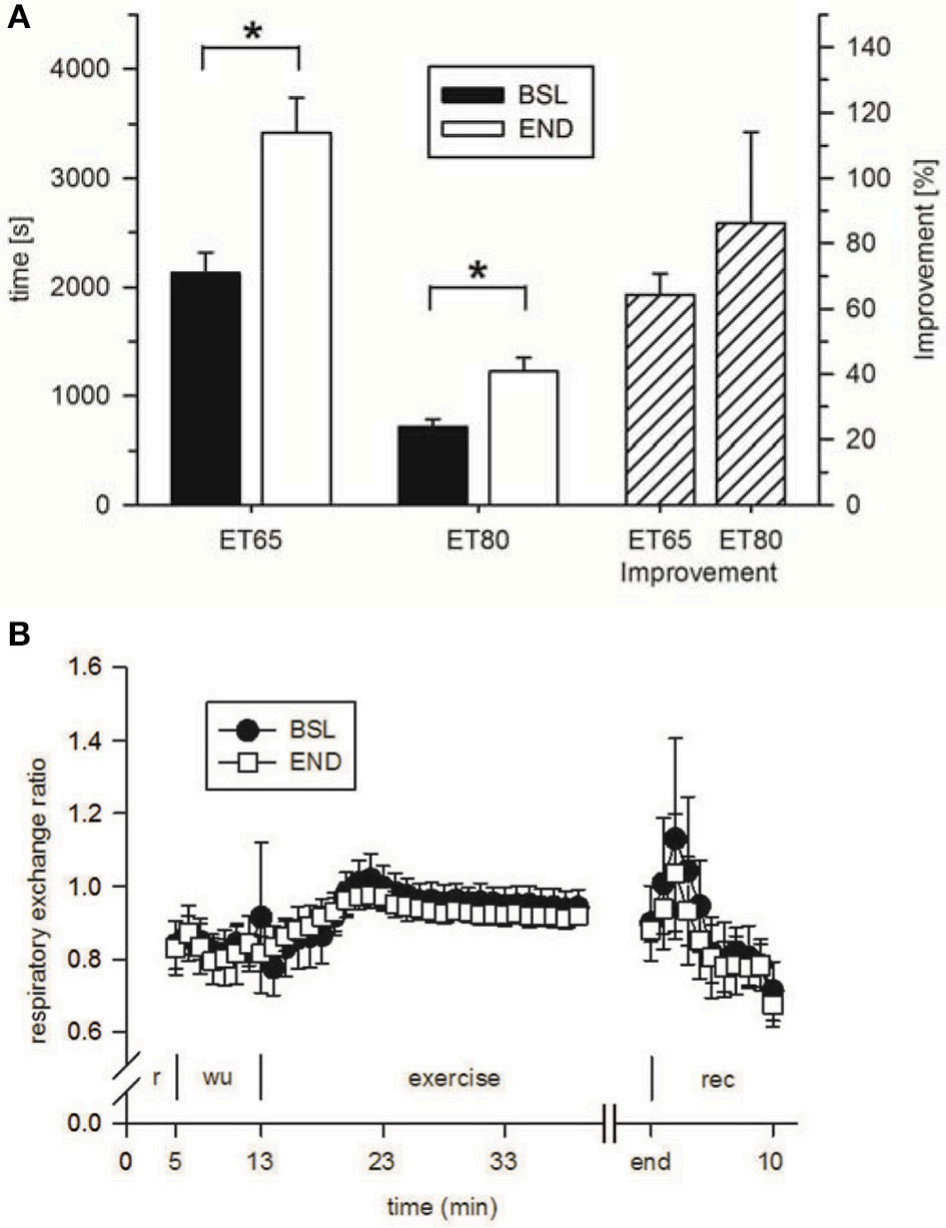
Analysis of **(A)** time-to-exhaustion in endurance tests at 65% P_max_ (ET65) and 80% P_max_ (ET80) at baseline (BSL) testing before the start of the HIHVT and at endpoint (END) testing after the entire HIHVT. Data are presented as means ± SE. ET65 and ET80 improvement: % improvement in time-to-exhaustion at END compared to BSL testing. ^*^Significantly different from BSL testing, *p* < 0.05. Analysis of **(B)** respiratory exchange ratio in endurance tests ET65 at BSL and END testing. Data are presented as means ± SE. Minutes 0–5: rest (r) on the cycle ergometer; min 6–13: 2 min running-in at 10 Watt (W) followed by 6 min warm-up (wu) at 30% maximum power (P_max_); endurance test to exhaustion at 80% P_max_ (exercise); recovery (rec); end: end of the test.

To analyze the effect of HIHVT on endurance capacity, time-to-exhaustion was evaluated in two endurance tests (ET), one with 65% maximum power (P_max_) (ET65) and the other one with 80% P_max_ (ET80). The eight subjects improved time-to-exhaustion by 64% in the ET65 and by 86% in the ET80 at END compared with BSL testing (Figure 6A, Table 3). The degree of improvement did not differ significantly between ET65 and ET80. These significant improvements in both ETs show that HIHVT led to increases in endurance capacity. During ET65 and ET80, $\dot{V}$O_2peak_, $\dot{VC}$O_2peak_ (Table 3) and RER (Figure 6B, ET80 data not shown) did not show significant differences.

**Table 3:** Performance, respiratory and metabolic parameters during or after various exercise tests.

	**IT**	**ET65**	**ET80**	**WT1**	**WT2**	**WT1**	**WT2**
	**P_peak_(W)**	**t_max_ (s)**	**t_max_ (s)**	**P_max_ (W)**	**P_max_ (W)**	**P_mean_ (W)**	**P_mean_ (W)**
BSL	308 ± 24	2130 ± 750	714 ± 252	1135 ± 66	779 ± 50	730 ± 32	527 ± 38
INT	320 ± 23^*^	ND	1044 ± 372	1079 ± 32	908 ± 66	763 ± 29	570 ± 38^*^
END	327 ± 22^*^	3420 ± 1212^*^	1224 ± 432^*^	1147 ± 65	885 ± 47	792 ± 42	580 ± 35^*^
	VO_2 peak_ (mmol^*^l^−1^)	VO_2 peak_ (mmol^*^l^−1^)	VO_2 peak_ (mmol^*^l^−1^)	VO_2 peak_ (mmol^*^l^−1^)	VO_2 peak_ (mmol^*^l^−1^)	VO_2 mean_ (mmol^*^l^−1^)	VO_2 mean_ (mmol^*^l^−1^)
BSL	3.71 ± 0.30	2.74 ± 0.25	3.16 ± 0.27	2.54 ± 0.27	1.78 ± 0.08	1.85 ± 0.19	1.69 ± 0.11
INT	3.84 ± 0.20	ND	3.56 ± 0.18	2.86 ± 0.36	2.09 ± 0.18	2.12 ± 0.27	1.90 ± 0.12
END	3.81 ± 0.20	2.81 ± 0.37	3.62 ± 0.17	2.33 ± 0.47	2.25 ± 0.23	1.73 ± 0.32	2.04 ± 0.18
	VCO_2 peak_ (mmol^*^l^−1^)	VCO_2 peak_ (mmol^*^l^−1^)	VCO_2 peak_ (mmol^*^l^−1^)	VCO_2 peak_ (mmol^*^l^−1^)	VCO_2 peak_ (mmol^*^l^−1^)	VCO_2 mean_ (mmol^*^l^−1^)	VCO_2 mean_ (mmol^*^l^−1^)
BSL	4.05 ± 0.22	2.74 ± 0.13	3.51 ± 0.15	2.93 ± 0.42	3.46 ± 0.24	2.03 ± 0.30	3.30 ± 0.16
INT	4.24 ± 0.21	ND	3.35 ± 0.15	3.16 ± 0.42	3.51 ± 0.25	2.22 ± 0.31	3.25 ± 0.23
END	4.25 ± 0.15	2.63 ± 0.12	3.41 ± 0.16	2.68 ± 0.57	3.78 ± 0.24	1.88 ± 0.38	3.45 ± 0.22
	Lactate_rest_ (mmol^*^l^−1^)	Lactate_rest_ (mmol^*^l^−1^)	Lactate_rest_ (mmol^*^l^−1^)	Lactate_rest_ (mmol^*^l^−1^)	Lactate_rest_ (mmol^*^l^−1^)		
BSL	0.85 ± 0.05	0.79 ± 0.08	1.02 ± 0.13	0.86 ± 0.06	NA		
INT	0.86 ± 0.11	ND	0.94 ± 0.12	0.72 ± 0.12	NA		
END	0.78 ± 0.10	0.84 ± 0.09	0.80 ± 0.10	0.83 ± 0.10	NA		
	Lactate_end_ (mmol^*^l^−1^)	Lactate_end_ (mmol^*^l^−1^)	Lactate_end_ (mmol^*^l^−1^)	Lactate_end_ (mmol^*^l^−1^)	Lactate_end_ (mmol^*^l^−1^)		
BSL	12.76 ± 1.11	8.46 ± 1.28	12.05 ± 1.20	5.57 ± 0.72	11.64 ± 0.62		
INT	12.00 ± 1.17	ND	12.90 ± 1.11	5.33 ± 0.83	12.70 ± 0.80		
END	12.87 ± 1.10	7.02 ± 1.35	12.09 ± 1.00	7.00 ± 0.64	13.31 ± 0.78		
	Glucose_rest_ (mmol^*^l^−1^)	Glucose_rest_ (mmol^*^l^−1^)	Glucose_rest_ (mmol^*^l^−1^)	Glucose_rest_ (mmol^*^l^−1^)	Glucose_rest_ (mmol^*^l^−1^)		
BSL	5.29 ± 0.15	5.31 ± 0.20	5.19 ± 0.21	5.11 ± 0.18	NA		
INT	5.14 ± 0.18	ND	5.41 ± 0.31	4.68 ± 0.17	NA		
END	5.41 ± 0.21	5.15 ± 0.13	5.61 ± 0.20	4.97 ± 0.16	NA		
	Glucose_end_ (mmol^*^l^−1^)	Glucose_end_ (mmol^*^l^−1^)	Glucose_end_ (mmol^*^l^−1^)	Glucose_end_ (mmol^*^l^−1^)	Glucose_end_ (mmol^*^l^−1^)		
BSL	4.73 ± 0.28	4.75 ± 0.29	4.44 ± 0.33	4.28 ± 0.17	4.30 ± 0.16		
INT	4.68 ± 0.12	ND	5.45 ± 0.44	4.70 ± 0.07	4.87 ± 0.07		
END	4.38 ± 0.22	5.04 ± 0.33	4.93 ± 0.32	4.55 ± 0.10	4.72 ± 0.16		
	Tri_rest_ (mmol^*^l^−1^)	Tri_rest_ (mmol^*^l^−1^)	Tri_rest_ (mmol^*^l^−1^)	Tri_rest_ (mmol^*^l^−1^)	Tri_rest_ (mmol^*^l^−1^)		
BSL	ND	1.09 ± 0.19	1.09 ± 0.07	1.29 ± 0.25	NA		
INT	ND	ND	1.63 ± 0.28	1.30 ± 0.21	NA		
END	ND	1.32 ± 0.18	1.25 ± 0.23	1.29 ± 0.23	NA		
	Tri_end_ (mmol^*^l^−1^)	Tri_end_ (mmol^*^l^−1^)	Tri_end_ (mmol^*^l^−1^)	Tri_end_ (mmol^*^l^−1^)	Tri_end_ (mmol^*^l^−1^)		
BSL	ND	1.21 ± 0.11	1.35 ± 0.08	1.43 ± 0.31	1.60 ± 0.39		
INT	ND	ND	1.75 ± 0.31	1.39 ± 0.27	1.49 ± 0.29		
END	ND	1.23 ± 0.19	1.51 ± 0.23	1.61 ± 0.29	1.61 ± 0.27		

Performance and respiratory parameters during and metabolic parameters at rest (rest) and at the end (end) of incremental test (IT), endurance tests at 65% P_max_ (ET65) and at 80% P_max_ (ET80), and Wingate tests 1 and 2 (WT1 and 2) at BSL, INT and END testing sessions. Data are presented as means ± SE. P_max_: maximum power; P _mean_, mean power; P_peak_, peak power; t_max_, time-to-exhaustion; Tri, triglycerides; NA, not applicable; ND, not determined.

* Significantly different from BSL testing, p < 0.05.

In incremental tests (ITs), subjects reached a P_peak_ of 308 ± 24 W at BSL testing, 320 ± 23 W at INT testing and 327 ± 22 W at END testing (Table 3). With 4.4% increase at INT testing and 6.9% increase at END testing both values are significantly higher than baseline results. The significant improvements in ITs indicate that the HIHVT led to increases in exercise performance. In ITs, $\dot{V}$O_2peak_ increased slightly but not significantly at INT and at END testing compared to BSL (Table 3), indicating that HIHVT did not markedly improve systemic aerobic capacity. In addition, $\dot{V}$CO_2peak_ did not change significantly between testing sessions.

To test for sprint and repeated sprint capability subjects performed a doubled Wingate Test (WT) meaning two 30 s all-out sprints (WT1 and WT2, respectively) with 1 min pause in between. P_max_ tended to be increased in WT2 at INT and END testing, but neither in WT1 and WT2 were significant changes due to the exercise program seen in P_max_ in (Table 3). Mean power (P_mean_) in WT1 also tended to be higher at INT and END testing compared to BSL testing but changes were not significant (Table 3). In WT2 P_mean_ was significantly increased at INT and at END compared to BSL testing (Table 3). The increase in P_mean_ from INT to END testing, however, was not significant. Results from the doubled WT indicate that the HIHVT led to enhanced sprint capability at least in WT2, which indicates an improved recovery between sprint tests.

No significant differences of $\dot{V}$O_2peak_ and $\dot{V}$O_2mean_ between testing sessions were detected in WT1 and 2 (Table 3), indicating no changes in whole body aerobic energy contribution. The same holds for $\dot{V}$CO_2peak_ and $\dot{V}$CO_2mean_.

#### Metabolic parameters

In ET65 and ET80 no significant changes in [Lac] were found between the testing sessions at the end of the tests (Table 3), indicating identical degrees of exertion.

Furthermore, no significant changes in [Glu] between the testing sessions were found at ET65 and ET80 at the end of the tests (Table 3), indicating no lasting HIHVT-induced changes in glucose handling.

**Figure 7:**
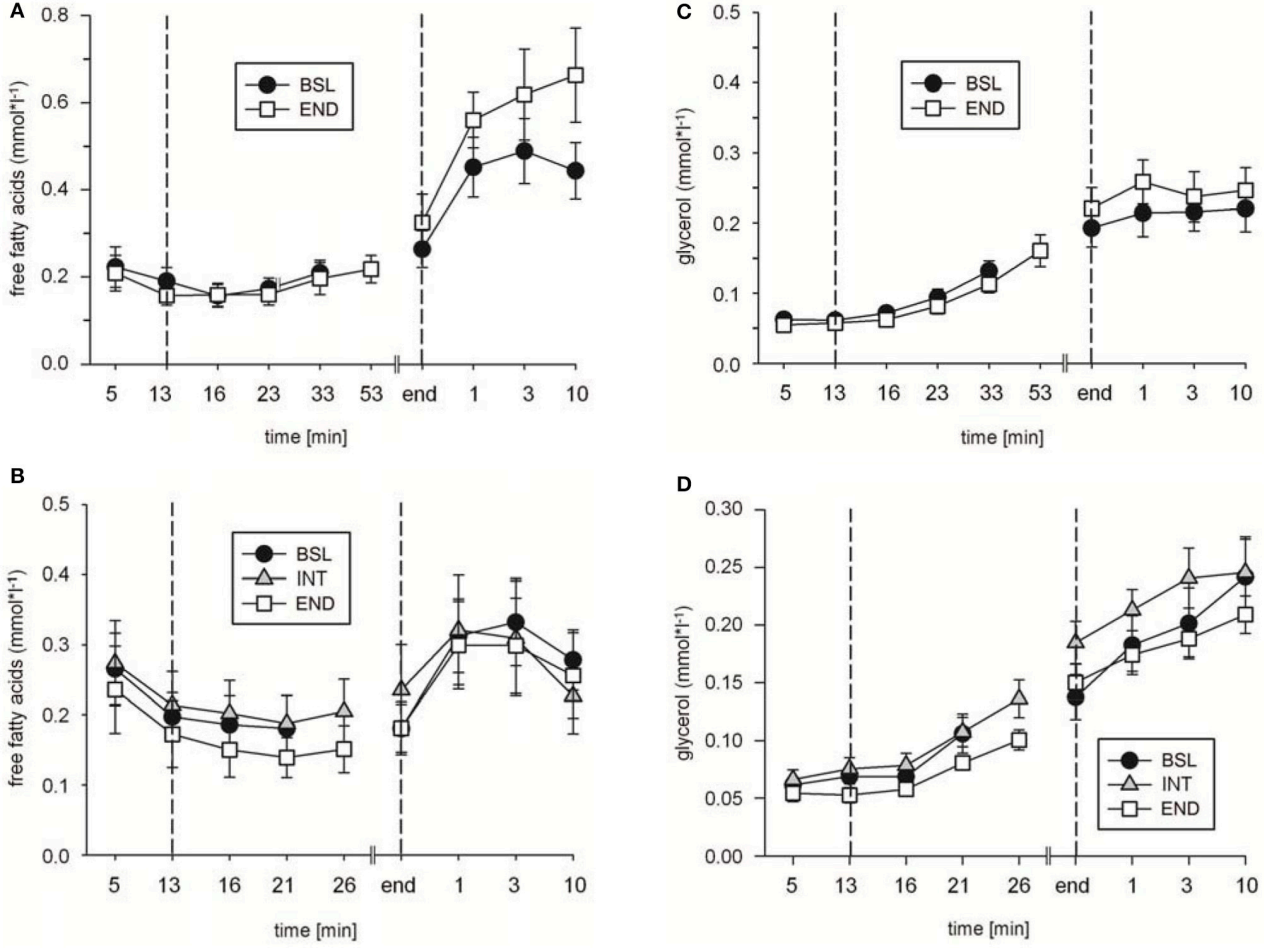
Analysis of **(A,B)** free fatty acid and **(C,D)** glycerol concentrations during **(A,C)** endurance tests (ET) 65, and **(B,D)** ET80 at baseline (BSL) testing, at endpoint (END) testing, and **(B,D)** as indicated, intermediate (INT) testing between the two sets of HIHVT training. Minutes 0–5: rest on the cycle ergometer; min 6–13: 2 min running-in at 10 Watt (W) followed by 6 min warm-up at 30% maximum power (P_max_); endurance test to exhaustion at **(A,C)** 65% and **(B,D)** 80% P_max_; recovery; end: end of the test.

In addition, in ET65 and ET80 as well as in WT no significant differences in [FFA] (Figures 7A,B; data not shown for WT), [Gly] (Figures 7C,D; data not shown for WT) and [Tri] (Table 3) were found between BSL, INT and END testing. Taken together, the data indicate no changes in lipid metabolism as a consequence of the training.

#### Hematocrit

**Figure 8:**
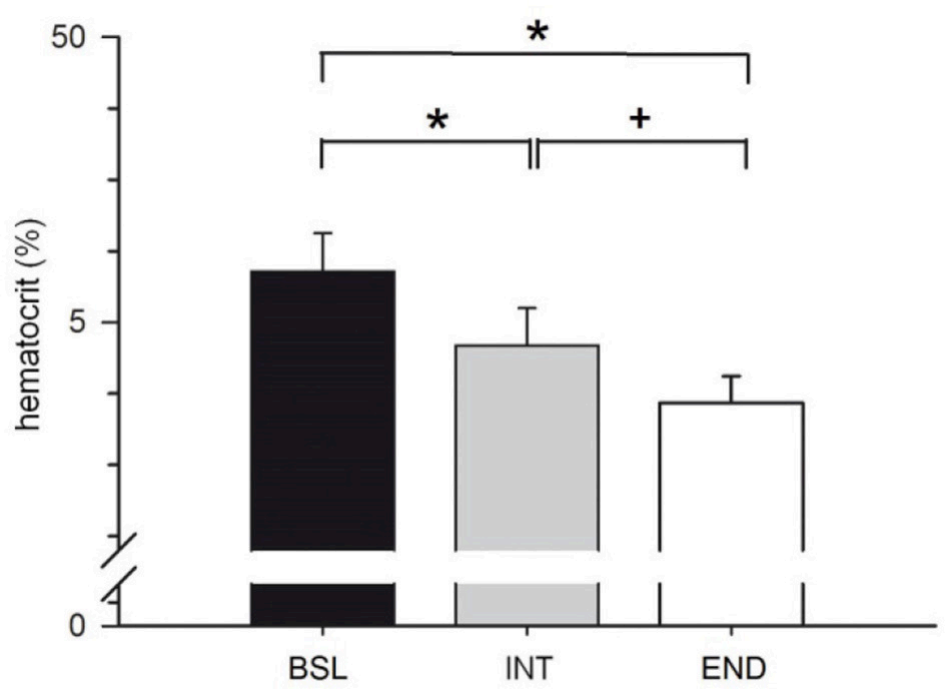
Analysis of hematocrit before start of the HIHVT at baseline (BSL) testing, at endpoint (END) testing, and at intermediate (INT) testing. Data are presented as means ± SE. ^*^Significantly different from BSL testing, *p* < 0.01; ^+^Significantly different from INT testing, *p* < 0.01.

Hematocrit was significantly decreased by 2.8% at INT compared to BSL testing and decreased by a further 2.2% at END compared to INT testing (Figure 8). The significantly lowered hematocrit (by 5% at END compared to BSL testing) indicates a significant expansion in plasma volume that may explain at least in part the reported improvements of endurance parameters.

### Fiber type-specific gene expression

To investigate possible effects of the HIHVT on fiber type-specific gene expression, we performed quantitative real-time PCR. We determined the mRNA expression levels of MHC isoforms I/β, IIa, and IId/x. The MHC isoforms are the hallmark of muscle fiber type. We also determined mRNA expression levels of PGC-1α and of PGC-1α-dependent citrate synthase (CS) and hydroxyacyl-CoA dehydrogenase (HADH) as markers of oxidative energy metabolism. Glyceraldehyde-3-phosphate dehydrogenase (GAPDH) served as a marker of glycolytic energy metabolism. We also investigated possible changes in the expression of the mitochondrial superoxide dismutase 2 (SOD2), a marker of antioxidant metabolism.

**Figure 9:**
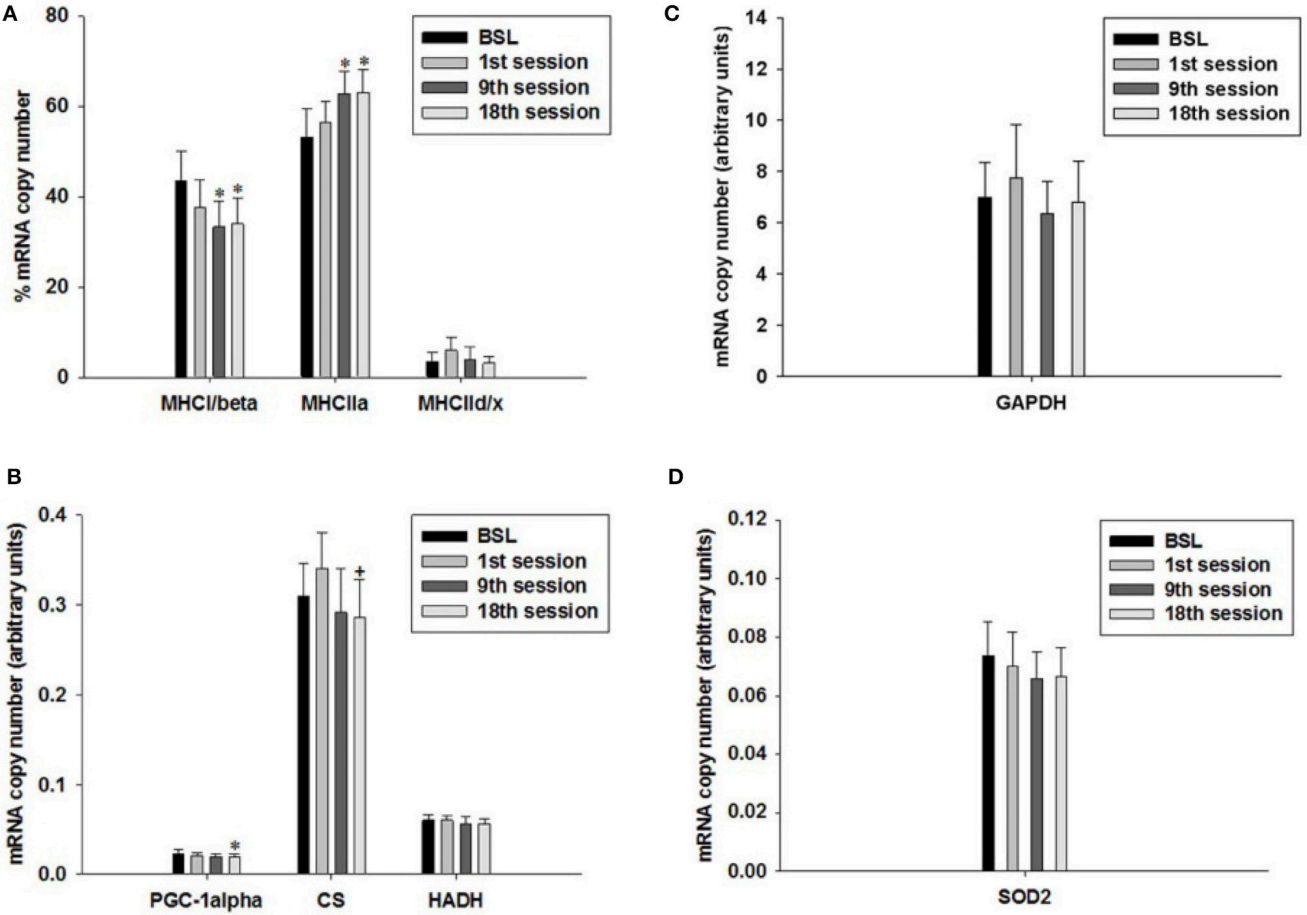
Quantitative real-time PCR (qPCR) analysis of mRNA expression of **(A)** myosin heavy chain (MHC) isoforms I/β, IIa, and IId/x, **(B)** peroxisome proliferator-activated receptor gamma coactivator 1α (PGC-1α), citrate synthase (CS) and hydroxyacyl-CoA dehydrogenase (HADH) as markers of oxidative energy metabolism, **(C)** glyceraldehyde-3-phosphate dehydrogenase (GAPDH) as a marker of glycolytic energy metabolism, and **(D)** mitochondrial superoxide dismutase 2 (SOD2) as a marker of radical metabolism. Muscle biopsies were taken before the start of the HIHVT at baseline (BSL) testing, after the first (1st), ninth (9th), and eighteenth (18th) training session. MHC isoform expression is shown as percentage of total MHC isoform mRNA copies (set to 100%). The mRNA expression of other marker genes is shown in arbitrary units. Data are presented as means ± SE. ^*^Significantly different from BSL testing, *p* < 0.05; ^+^Significantly different from the first training session, *p* < 0.05.

The percentage of transcript expression of MHCI/β, IIa and IId/x did not change after the first training session compared to the BSL mRNA percentage (Figure 9A). In contrast, the percentage of MHCI/β mRNA decreased after the ninth and eighteenth training session (Figures 1, 9A), while the percentage of MHCIIa mRNA increased. The percentage of MHCIId/x mRNA remained unchanged. Therefore, the HIHVT induced a MHC isoform shift from slow to fast on the level of mRNA expression.

The level of PGC-1α mRNA expression did not change after the first and after the ninth training session, but decreased after the eighteenth training session, when compared to BSL mRNA level (Figure 9B). Furthermore, the mRNA expression levels of CS were significantly lower at the end of the eighteenth compared to the end of the first training session (Figure 9B). Moreover, we found an excellent overall correlation between the values of PGC1α and CS (*p* < 0.001; not shown). The two changes, together with the decrease of MHCI/β mRNA expression after the ninth and eighteenth training session, support the idea of a HIHVT-induced slow-to-fast fiber type transformation on the mRNA level, which includes MHC as well as metabolic changes. However, mRNA expression levels of HADH did not change significantly after the first or after the ninth and eighteenth training session (Figure 9B). Unchanged mRNA levels of this latter marker of oxidative energy metabolism are at variance with the decrease of PGC-1α and CS mRNA expression observed after the eighteenth training session and would not be in agreement with a classical slow-to-fast fiber type transformation. The same holds true for the unchanged transcript expression of GAPDH and SOD2 seen after the first, ninth and eighteenth training session (Figures 9C,D). Taken together, the lack of effects of HIHVT on mRNA expression of HADH, GAPDH and SOD2 appear inconsistent with the effects on MHC isoform, CS and PGC-1α mRNA expression.

Nevertheless, our results can be put into a different perspective when parameter levels in single training subjects are considered. Seven of eight subjects showed a decrease of PGC-1α mRNA expression after the ninth training session as compared to the BSL mRNA level, while no significant change was found for the whole cohort (Table 4, Figure 9B). After the eighteenth training session, all eight subjects showed a decrease in PGC-1α mRNA expression, thus for the whole group a significant change compared to the BSL testing level was obtained. The difference between the ninth and eighteenth training session may be due to the fact that the number of training subjects was fairly low and the training history was different among the subjects at the start of the study. Similarly, with regard to CS and HADH, at least 75% of all subjects showed a decrease of mRNA expression levels after the ninth and eighteenth training session, while the whole cohort showed a significant change compared to the BSL mRNA level only in the case of CS after the eighteenth training session (Table 4, Figure 9B). Therefore, considering single subject data, the changes of mRNA expression of all markers of oxidative energy metabolism employed show the tendency to be compatible with the changes in MHC isoform mRNA expression. Taken together, expression data of markers of energy metabolism and MHC isoforms indicate a nearly complete HIHVT-induced slow-to-fast fiber type transformation on the mRNA level, with the exception of the marker of glycolytic energy metabolism. Nonetheless, the demonstrated effects of HIHVT on parameters of exercise performance, especially endurance capacity, clearly cannot be explained by the presented mRNA level measurements of markers of energy metabolism or of SOD2.

**Table 4:** Changes in mRNA expression of markers of oxidative energy metabolism in single subjects and in the cohort.

	**Single subject**	**Single subject**	**Cohort**	**Cohort**
**mRNA level**	**9th session**	**18th session**	**9th session**	**18th session**
PGC-1alpha	7/8 decreased	8/8 decreased	n.s.	*p* < 0.05
CS	6/8decreased	6/8 decreased	n.s.	*p* < 0.05
HADH	6/8decreased	7/8 decreased	n.s.	n.s.

*Number of subjects relative to total number of subjects (8) with a decrease in mRNA expression level of indicated oxidative energy marker genes after the ninth (9th) and eighteenth (18th) training session as compared with significances of the respective changes in the cohort*.

## Discussion

### Changes in muscular parameters

In the present paper the effects of a 6-s sprint HIHVT consisting of two sets of sessions of very short high intensity cycle ergometer sprints on parameters of skeletal muscle fiber type, exercise parameters, and metabolites were demonstrated. A slow-to-fast shift in MHC expression as demonstrated by an increase in MHCIIa, and a decrease in MHCI/β mRNA expression was induced by the HIHVT already after the first set of training sessions. The shift in MHC expression was accompanied by a decreased expression—or a trend toward decreased expression—of markers of oxidative energy metabolism, while plasma levels of lipid metabolites remained unchanged. Performance parameters like time-to-exhaustion in ET65% and ET80% as well as P_peak_ in IT and P_mean_ and P_max_ in WT2 were all improved by the present HIHVT, indicating improvements in repeated sprint ability and endurance performance. Moreover, [Lac] decreased significantly in ET65, and in ET80 there is a tendency to lower [Lac]. However, $\dot{V}$O_2peak_ was not significantly altered during ITs. Sprint interval trainings as presented here are expected to be a potent and time-efficient means to improve endurance exercise performance (Coyle, 2005). Indeed, the data of the present study provide another example for the potency and efficiency of intense interval training in terms of endurance exercise parameters. It produces training effects comparable to those induced by endurance training. The heterogeneous training history of the group of subjects becomes apparent in the good correlation we find between the exercise-induced changes in ET80% (ΔET80%) and the initial ET80%, where ΔET80% decreases with increasing initial ET80% (*r* = 0.69; *P* < 0.05). The relation was similar for ET60%, although not significant. Similarly, ΔP_peak_ decreased significantly with increasing initial P_peak_ value (*r* = 0.76; *P* < 0.05). This indicates an influence of training status on the size of the training effects. Nevertheless, in spite of the rather small sample size and the heterogeneity of the samples, many parameters changed statistically significantly for the whole cohort by the HIHVT protocol.

An outcome very similar to the present one has been demonstrated earlier after a running sprint HIT by Dawson et al. (1998), who reported a nearly complete slow-to-fast transformation that is reflected in an increase in the proportion of type II fibers, a decrease in citrate synthase activity but no change in the glycolytic marker phosphofructokinase. This was accompanied by improved sprint and endurance performance parameters. This study, like ours, suggests that HIT can induce an almost complete slow-to-fast fiber transformation. Several other related studies reported deviating results: (1) Jansson et al. (1990) in a high intensity sprint training study also showed decreases in type I and increases in type IIA MHC, but did not find changes in performance parameters as assessed by WTs. (2) Sprint training of an especially high intensity has interestingly been demonstrated to lead to an additional conversion of type IID to type IIA fibers (Esbjörnsson et al., 1993), which would indicate that the outcome of high intensity sprint training may be related to the total training volume. (3) In line with this latter paper, another highly intense strength and interval training has also demonstrated a shift toward an increased fraction of type IIA and decreases in the number of type I and IID fibers (Andersen et al., 1994). The two last-mentioned studies did not present metabolic data. We conclude that with regard to changes in fiber type and/or MHC pattern, the present results are partly similar to those of some previous investigations.

This is not the case with regard to the study of Linossier et al. (1993). The sprint training used by Linossier et al. (1993) and the present 6-s sprint HIHVT have a similar design. However, the total work performed during the sessions in our investigation is about twice as high as that used in the study of Linossier et al. (1993). This is mainly due to the larger number of intervals (90 vs. 16 to 30) and shorter breaks between exercise bouts (24 vs. 55 s). Linossier et al. (1993) found an increase in the number of type I and a decrease in type IID fibers, while the number of type IIA fibers remained unaltered. Therefore, the effects on fiber type and MHC expression, respectively, are contradictory between the two studies with regard to the direction of the shift. The impact on the oxidative energy metabolism is also different, with reduction or a trend toward reduction of the expression of oxidative markers CS and HADH in our study, vs. no change in enzymatic activities of the same markers in the study of Linossier et al. (1993). The effects on markers of glycolytic energy metabolism were also different, with an increase of phosphofructokinase and lactate dehydrogenase activities (and no change of hexokinase activity) seen by Linossier et al. (1993), but no change of GAPDH seen in our study. It is not clear whether differences in the study design and/or in the determination of muscle fiber type parameters can be the underlying reason for the different outcomes of the two studies.

The oxidative capacity of type IIA fibers is known to be lower than that of type I fibers (Saltin et al., 1977). Decreases in PGC-1α-dependent markers of oxidative energy metabolism have been observed here in a majority of individual training subjects after the first and second set of training sessions. This indicates a trend toward a reduction of muscular oxidative capacity induced by the present training protocol albeit not all parameters reach statistical significance in the whole cohort. This interpretation of our data is strongly supported by the decreased PGC-1α mRNA levels observed after the second set of training sessions and suggests a trend toward a nearly complete slow-to-fast-transformation on the mRNA level, with the exception of glycolytic energy metabolism. Decreased muscular oxidative capacity and the lack of changes in plasma lipid metabolite levels suggest that observed improvements in endurance performance parameters are likely to be caused by other factors. Unchanged lipid metabolism is also reflected by the lack of significant differences in RER during ET65 and ET80. The effects of HIHVT are also not explainable by changes in SOD2 transcription. Reported effects of N-acetylcysteine infusion prior to or during exercise have led to the hypothesis that reactive oxygen species (ROS) might play a role in exercise performance (McKenna et al., 2006). The present study shows no up-regulation of SOD2, and thus a possible role of ROS in mediating the effects of HIHVT is not apparent from the data presented. Nevertheless, it cannot be excluded that the expression of other enzymes involved in ROS detoxification might be changed during the HIHVT.

### Changes in metabolite and blood parameters

Fiber type alone is not the only determinant of athletic performance (Zierath and Hawley, 2004). At least in athletes, distance running performance is most strongly related to $\dot{V}$O_2max_, but only modestly to fiber composition (Foster et al., 1978). Furthermore, this latter study showed that there is only a weak relationship between oxidative marker succinate dehydrogenase (SDH) activity and either performance or $\dot{V}$O_2max_. Indeed, other studies demonstrated performance improvements in response to HIT without a change in CS enzyme activity (Weston et al., 1997; Cochran et al., 2014). We now could demonstrate improvements in endurance exercise parameters despite a partly significant tendency toward a decrease in muscular oxidative capacity in HIHVT. In contrast to this, some high intensity sprint interval training studies have even demonstrated increases in CS activity (Burgomaster et al., 2006, 2008; Larsen et al., 2015).

At first glance, decreased [Lac] during training seems to be at odds with the finding of a decrease in muscular oxidative together with unaltered glycolytic capacity. While changes seen in the biopsy sample are likely not representative for the entire working musculature, it is in addition well-known that alterations of whole body metabolism and muscular oxidative capacity are not necessarily linked. This has been demonstrated in an endurance exercise study (Green et al., 1992), which showed that training resulted in pronounced reductions in glycogen utilization that were accompanied by reductions in muscle lactate concentrations. At the same time, the activities of two enzymes of muscular oxidative energy metabolism (CS and SDH) remained unaffected. In line with this finding, a close relationship between blood lactate and glycogen utilization has been demonstrated in a high intensity cycle ergometry study (Coyle et al., 1988). Interestingly, both factors varied in this study by up to a factor of two among cyclists with similar $\dot{V}$O_2_max, pointing to significant differences in individual metabolic responses to the same training regime. Improved clearance of lactate by the liver, increased lactate utilization by the heart and slow muscle fibers and perhaps a larger distribution volume for lactate might explain the reduction of [Lac]. Indeed, [Lac] has been shown to negatively correlate with endurance performance during submaximal exercise (Coyle et al., 1988). It has also been shown that skeletal muscle buffering capacity is affected by high-intensity, submaximal interval training, which indicates another potentially important factor determining endurance performance (Weston et al., 1997).

The observed reduction of hematocrit in the present study indicates an increase in blood volume. Hypervolemia accompanied by a reduction of blood viscosity is a well-documented effect of endurance exercise training (Schmidt et al., 1988; Convertino, 1991; Fellmann, 1992). Interestingly, the effects of exercise on blood volume and viscosity may differ depending on the type and intensity of exercise (Neuhaus and Gaehtgens, 1994; El-Sayed et al., 2005). Several factors are thought to contribute to the effects of hypervolemia and reduced blood viscosity on exercise performance. Hypervolemia may be advantageous for thermoregulation by providing more fluid to increase skin perfusion for heat dissipation. Hypervolemia also can positively affect cardiac output via increased stroke volume by providing larger vascular volume and filling pressure. Furthermore, a reduced resistance as a result of decreased blood viscosity may facilitate oxygen delivery to the exercising muscle by improving perfusion and reduce the work required by the heart. Further investigations are required to clarify the mechanisms underlying the improvements in endurance parameters observed in the present study.

In conclusion, the 6-s sprint HIHVT led to a shift toward increased MHCIIa and decreased MHCIβ expression and a trend toward decreased muscular oxidative capacity, but no significant change in the glycolytic marker. Thus, HIHVT induces two major constituents of a complete slow-to-fast fiber type transformation at least on the mRNA level. The findings reported here fully support one earlier study by Dawson et al. (1998), but are to different extents at variance with many previous findings (Jansson et al., 1990; Esbjörnsson et al., 1993; Linossier et al., 1993; Andersen et al., 1994; Weston et al., 1997; Burgomaster et al., 2006, 2008; Cochran et al., 2014; Larsen et al., 2015). The biological response, Dawson et al. (1998) and we observe upon sprint training, namely a slow-to-fast fiber transformation, seems logical and in line with many well-documented observations in animals (Pette and Vrbova, 1992). Nevertheless, the improvements in endurance exercise performance demonstrate that the 6-s sprint HIHVT at the same time is an effective and time-efficient training regimen to induce training effects comparable to those of aerobic endurance training. Since the changes seen in skeletal muscle cannot explain the increased endurance exercise performance, other mechanisms are likely to contribute to these improvements. Indeed, it has been shown that plasma volume expansion alone improves endurance performance (Berger et al., 2006).

## Ethics statement

This study was carried out in accordance with the recommendations of the Ethik-Kommision der Medizinischen Hochschule Hannover with written informed consent from all subjects. All subjects gave written informed consent in accordance with the Declaration of Helsinki. The protocol was approved by the Ethik-Kommision der Medizinischen Hochschule Hannover.

## Author contributions

JE, NM, GG, and JM: concept of study; JE, JF, and NM: exercise testing and blood parameters; MM and SE: biopsy sampling; JM: mRNA measurements; JE, NM, GG, and JM: writing of paper.

### Conflict of interest statement

The authors declare that the research was conducted in the absence of any commercial or financial relationships that could be construed as a potential conflict of interest.
